# Combined effects of mild hypothermia and nitrous-oxide-induced narcosis on manual and cognitive performance

**DOI:** 10.1152/ajpregu.00246.2023

**Published:** 2024-01-08

**Authors:** Maaike I. Moes, Antonis Elia, Mikael Gennser, Michail E. Keramidas

**Affiliations:** Division of Environmental Physiology, Swedish Aerospace Physiology Center, KTH Royal Institute of Technology, Stockholm, Sweden

**Keywords:** cold adaptation, diving, inert gas narcosis, memory, nonassociative learning

## Abstract

Divers are at enhanced risk of suffering from acute cognitive deterioration because of the low ambient temperatures and the narcotic action of inert gases inspired at high pressures. Yet, the behavioral effects of cold and inert gas narcosis have commonly been assessed in isolation and during short-term provocations. We therefore evaluated the interactive influence of mild hypothermia and narcosis engendered by a subanesthetic dose of nitrous oxide (N_2_O; a normobaric intervention analog of hyperbaric nitrogen) on cognitive function during prolonged iterative exposure. Fourteen men partook in two ∼12-h sessions (separated by ≥4 days), wherein they performed sequentially three 120-min cold (20°C) water immersions (CWIs), while inhaling, in a single-blinded manner, either normal air or a normoxic gas mixture containing 30% N_2_O. CWIs were separated by a 120-min rewarming in room-air breathing conditions. Before the first CWI and during each CWI, subjects performed a finger dexterity test, and the Spaceflight Cognitive Assessment Tool for Windows (WinSCAT) test assessing aspects of attention, memory, learning, and visuospatial ability. Rectal and skin temperatures were, on average, reduced by ∼1.2 °C and ∼8 °C, respectively (*P* < 0.001). Cooling per se impaired (*P* ≤ 0.01) only short-term memory (∼37%) and learning (∼18%); the impairments were limited to the first CWI. N_2_O also attenuated (*P* ≤ 0.02) short-term memory (∼37%) and learning (∼35%), but the reductions occurred in all CWIs. Furthermore, N_2_O invariably compromised finger dexterity, attention, concentration, working memory, and spatial processing (*P* < 0.05). The present results demonstrate that inert gas narcosis aggravates, in a persistent manner, basic and higher-order cognitive abilities during protracted cold exposure.

## INTRODUCTION

During exposure to cold, basic and higher-order cognitive abilities are often disturbed. For instance, in mildly hypothermic individuals, manual dexterity and vigilance are impaired, reaction time is extended, and accuracy, attention, short-term memory, and decision-making are compromised (for reviews, see Refs. 1–3). These cold-induced neurocognitive impairments, which exhibit a large interindividual variability, have been ascribed to the depression of synaptic transmission and of central and peripheral nerve conduction velocity (4–6), the reduction in cerebral blood flow and oxygen (O_2_) delivery (7), and/or, perhaps, the desensitization of catecholamine release in response to mental demands (8, 9). The magnitude of hedonic perception engendered by cold stress appears to also contribute to the cognitive decline, via a “distraction” effect; that is, thermal discomfort disturbs and diverts attention away from the required cognitive task (10). Yet, regardless of the underlying mechanisms, the impact of cold on cognition has primarily been assessed during short-term (i.e., ≤3 h; cf. Ref. 1) provocations, whereas information on protracted exposure to cold is scarce (e.g., Ref. 83), especially in uncompensable (i.e., high heat loss) thermal conditions.

Divers are at enhanced risk of suffering from acute cognitive deterioration, not only because of the low ambient temperatures encountered underwater (11–14), but also because of the independent action of the inspired inert gases on the central nervous system (15, 16). Thus breathing air or nitrogen (N_2_)-O_2_ mixtures at high [>3 atmospheres absolute (ATA)] pressures causes N_2_ narcosis, which is described, inter alia, by euphoric feelings, psychomotor disturbances, poor concentration, disrupted neuromuscular coordination, impaired judgment, diminished memory, and spatial disorientation (17–20).

At atmospheric pressure, inhalation of a subanesthetic dose of nitrous oxide (N_2_O) appears to exert a similar influence on cognition and behavior (21–25) and hence has commonly served as a normobaric intervention analog to breathing compressed air or high-N_2_ gas mixtures at depths (23, 26–30). Human-based studies have demonstrated that behavioral tolerance to N_2_O does not develop, given that its deleterious effects on cognition fail to subside over prolonged and/or repeated N_2_O administration (31–33). Notably, normobaric N_2_O, as well as hyperbaric N_2_, also perturbs autonomic and behavioral thermoregulation to cold; namely, shivering thermogenesis and thermoperception are suppressed consistently (34–38). However, to our knowledge, the manner in which inert gas narcosis interferes with cognitive processes during cold stress has as yet not been determined.

Accordingly, in this study, we tested the hypothesis that N_2_O-induced narcosis would aggravate the neurocognitive impairments provoked by cold stress. We also sought to evaluate whether the interactive influence of N_2_O and cold on cognition would prevail or gradually dissipate over prolonged iterative exposure. To address these questions, a within-subject design was employed, wherein finger dexterity and aspects of attention, memory, learning, and visuospatial processing were assessed regularly during the course of three 20°C water immersions, performed sequentially within a ∼10-h period, while subjects were breathing either normal air or a normoxic gas mixture containing 30% N_2_O. Although it might be argued that sustained cold-water immersion would have increased the ecological validity of the study, we used immersions in 20°C water in a repeated fashion to evoke levels of deep body cooling (∼1°C) safely and relatively rapidly that are commonly encountered by divers conducting protracted (≤9 h) cold dives with wet or dry suits (12, 13, 39, 40). We anticipated that N_2_O would compromise, in a persistent manner, the motor and cognitive abilities.

## METHODS

### Ethics Approval

The study was performed at the Division of Environmental Physiology, Royal Institute of Technology (Solna, Sweden). The experimental protocol was approved by the Regional Human Ethics Committee in Stockholm (Reference No. 2021-05314-01) and conformed to the Declaration of Helsinki (except for registration in a database). Subjects were informed about the experimental procedure before giving their written consent to participate. This study is part of a larger project examining the effects of N_2_O on human thermoregulation to cold. A subset of the temperature, thermoperceptual, and cardiorespiratory data have been presented and discussed previously (see Ref. 38); however, the data concerning the neurocognitive and psychomotor performance are novel.

### Subjects

Fourteen healthy men [mean (range) age: 23 (18–28) years; height: 179 (164–192) cm; and weight: 71.4 (55.7–93.6) kg] participated. An a priori power analysis was not performed for the specific data set, because the present work addressed a secondary purpose of the larger project (see Ref. 38). All subjects resided in the Stockholm region for ≥6 mo before the study, were physically active, were nonsmokers, and were not taking any prescription medications. None was engaged in diving-related activities. The following exclusion criteria were applied: *1*) history of cold injury, *2*) history or family history of vitamin B_12_ deficiency, *3*) habitual use of N_2_O, *4*) complete abstinence from alcohol, and *5*) regular exposure to severe cold water (e.g., winter bathing or swimming). Twelve subjects were right-handed; none was ambidextrous.

### Experimental Protocol

**Figure 1.**
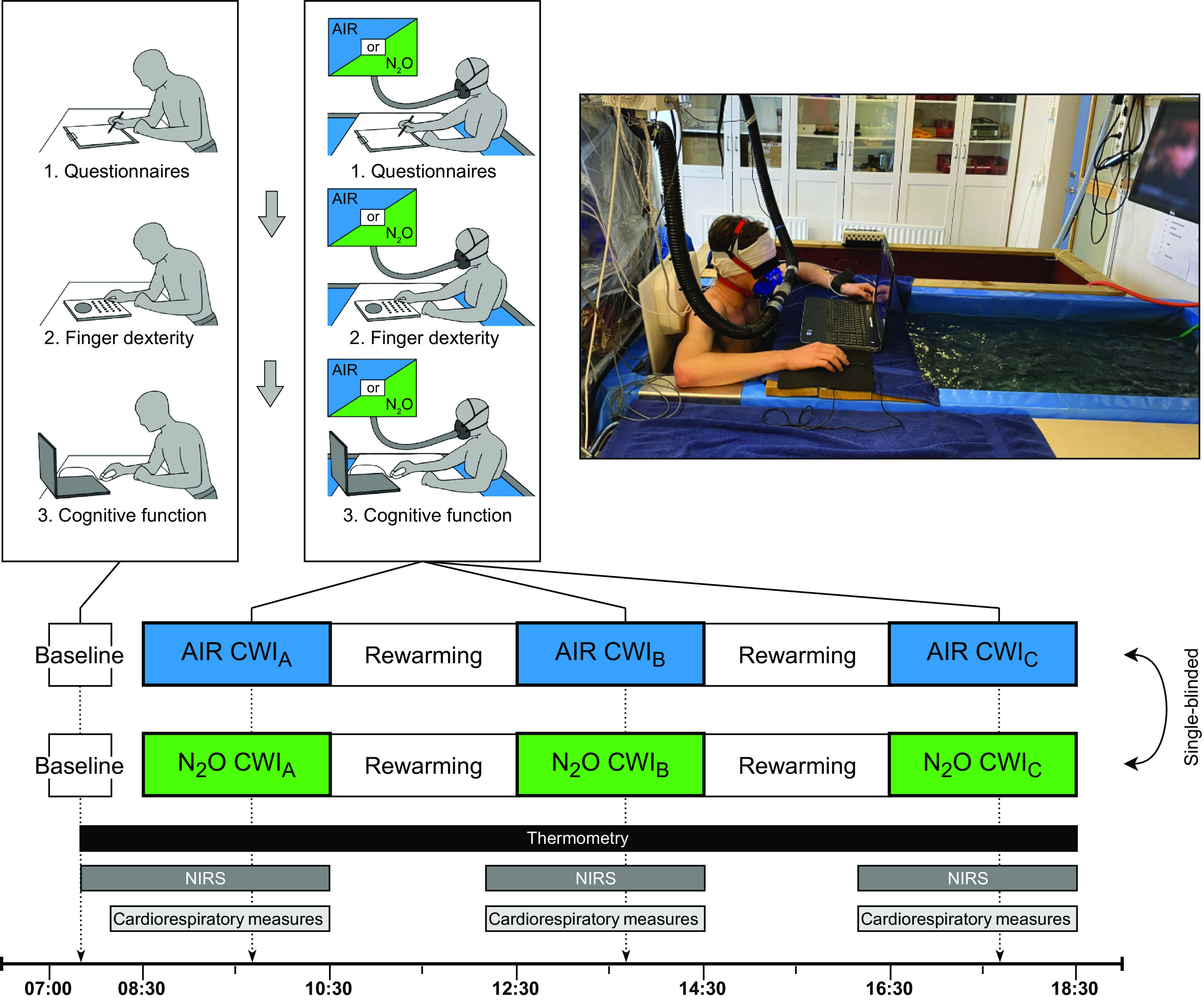
Overview of the experimental protocol of the two ∼12-h test sessions, during which subjects were breathing either normal air or a normoxic gas mixture containing 30% N_2_O. AIR, normal air; CWI_A_, 1st cold-water immersion; CWI_B_, 2nd cold-water immersion; CWI_C_, 3rd cold-water immersion; NIRS, near-infrared spectroscopy; N_2_O, nitrous oxide.

Subjects visited the laboratory on three separate occasions: a ∼4-h preliminary session and two main ∼12-h test sessions (Fig. 1). During the preliminary session, medical screening and anthropometric measures were performed. Subjects were also familiarized with the equipment and experimental procedure. Specifically, they were requested to perform six times, interspersed by a ≥5-min interval, the finger dexterity and the cognitive function tests (see *Measurements and Instrumentation*).

Subjects underwent sequentially three ≤120-min cold (20°C) water immersions (CWIs; first CWI: CWI_A_; second CWI: CWI_B_; and third CWI: CWI_C_) while they were breathing, in a single-blinded manner, either normal air (AIR) or a normoxic gas mixture containing 30% N_2_O. The specific partial pressure of N_2_O at atmospheric pressure exerts a similar behavioral influence as does the partial pressure of N_2_ in air at 6 ATA (22, 41). CWIs were separated by a 120-min rewarming period, during which subjects were inspiring room air, and their rectal temperature (T_rec_) returned to the CWI_A_ preimmersion values (see Ref. 38). In both test sessions, subjects reported to the laboratory at 07:00; CWI_A_, CWI_B_, and CWI_C_ were performed at ∼8:30, 12:30, and 16:30 h, respectively. For the individual subject, the AIR and N_2_O sessions were carried out within a 5-wk period and were separated by at least 4 days. The order of the sessions was alternated among subjects: six participated first in the AIR session and eight in the N_2_O session. At the beginning of the sessions and during each rewarming phase, subjects ate a small snack (∼180 kcal) and drank ∼250 mL water. Subjects were always dressed in regular swim shorts. The temperature and relative humidity in the laboratory were ∼27°C and ∼40%, respectively.

Each session commenced with a baseline phase, wherein subjects were seated comfortably in a chair and were breathing room air. They were asked initially to complete two questionnaires: the Profile of Mood States-Short Form (POMS-SF) and the Multidimensional Fatigue Inventory (MFI). Then, a 3-min resting period ensued, during which the baseline temperature and cerebral-oxygenation responses were monitored. Thereafter, subjects performed a 3-min finger dexterity test, and, after a ∼1-min interval, the cognitive function test. Subjects were then transferred to a gurney and assumed a semireclined position; they were equipped with an oronasal mask and breathed, through a low-resistance, two-way respiratory valve, either AIR or N_2_O. The inspiratory side of the valve was connected via respiratory corrugated tubing to a bag filled with the premixed humidified breathing gas. After a 20-min recording of the preimmersion respiratory and cardiovascular values (this phase preceded all 3 CWIs), subjects were immersed up to the level of the xiphoid process into a tank, filled with 20°C water, for 120 min (note: CWI was terminated prematurely if T_rec_ dropped below 35°C). The temperature of the water was monitored continuously with two thermistors (PT100, Texas Instruments, Dallas, TX) and, if necessary, was adjusted by adding cold or warm water. Throughout CWIs, subjects remained in a semiupright sitting position, with both arms being supported at the level of the heart above the water surface. At the 70th minute (or earlier, if the body core cooling rate was accelerated, indicating premature termination) of each CWI, subjects were requested to repeat, in the same sequence, the tests that were performed during the baseline phase: they initially completed the POMS-SF and MFI and then conducted the finger dexterity test and finally the cognitive function test. During CWIs, subjects were free to watch movies; yet, during the psychometric and neurocognitive measurements, the movie was paused. No feedback was provided to the subjects at any time point of the testing.

### Measurements and Instrumentation

T_rec_ was monitored using a rectal thermistor (Yellow Springs Instruments, Yellow Springs, OH), inserted ∼10 cm beyond the anal sphincter. Mean skin temperature (T_sk_) was derived from the unweighted average of skin temperatures, recorded with copper-constantan (T-type) thermocouple probes (Physitemp Instruments, Inc., Clifton, NJ), placed at either the left (in the right-handed subjects) or the right (in the left-handed subjects) side of body, on the following sites: foot, calf, thigh, abdomen, upper arm, forearm, index finger, and forehead. The index finger skin temperature (T_finger_) of the nondominant hand obtained during the period of the dexterity test was also reported separately. Mean body temperature (${\bar{\text{T}}}_{\text{b}}$) was calculated by the equation: ${\bar{\text{T}}}_{\text{b}}$ = 0.64 × T_rec_ + 0.36 × T_sk_ (42). Inspiratory minute volume (V̇_I_) was measured with a turbine ventilation module (KL Engineering, Los Angeles, CA). Expired O_2_ (Applied Electrochemistry model S-3A/I, Pittsburgh, PA) and carbon dioxide (Beckman Model LB-2, Fullerton, CA) concentrations were analyzed from a 10-l Plexiglas mixing box, and oxygen uptake (V̇o_2_) was calculated with the Haldane transformation. Beat-to-beat arterial pressures were measured using a volume-clamp technique (Finometer, Finapres Medical Systems, Amsterdam, The Netherlands), with the pressure cuff placed on the middle finger of the nondominant hand, and with the reference pressure transducer positioned at the level of the heart. Heart rate (HR) was derived from the arterial pressure curve as the inverse of the interbeat interval. Cerebral oxygenation was monitored using a three-wavelength (735, 810, and 850 nm) near-infrared spectroscopy (NIRS) device (NIRO-200NX, Hamamatsu Photonics, Japan). The probe was positioned, at a fixed interoptode distance of 4 cm, over the left prefrontal cortex at the midpoint between Fp1 and F3 landmarks of the international electroencephalogram 10–20 system. The modified Beer-Lambert law was used to determine concentration changes in oxygenated (Δ[O_2_Hb]) and deoxygenated (Δ[HHb]) hemoglobin; total hemoglobin (Δ[tHb]) was calculated as the sum of Δ[O_2_Hb] and Δ[HHb] (43). The NIRS signal was sampled at 1 Hz and expressed relative to the 3-min period preceding the baseline dexterity test.

The POMS-SF and MFI questionnaires were presented in a hardcopy format and were replied to within a <10-min period. Subjects were instructed to respond based on how they felt at that moment in time. POMS-SF is a 37-item questionnaire assessing six specific mood states: tension, depression, anger, vigor, fatigue, and confusion (44). The description of the subjects’ feelings was provided based on a 5-point Likert Scale from 0 (“not at all”) to 4 (“extremely”). The total mood disturbance was also calculated; wherein the higher score represents a greater mood disturbance (45). MFI is a 20-item self-rating tool evaluating five subscales of fatigue: general fatigue, physical fatigue, reduced activity, reduced motivation, and mental fatigue (46). Each subscale contains four items and the answer ranges from 1 (“yes, that is true”) to 5 (“no, that is not true”); the higher score in each subscale (ranges from 4 to 20) indicates a greater fatigue sensation.

Finger dexterity was assessed with the O’Connor pegboard (model 32021, Lafayette Instruments, Lafayette, IN). Subjects were requested to, by using only their dominant hand, place three pins in as many pinholes as possible, within a 3-min period (84). They were instructed to fill the holes row-by-row and from left-to-right when right-handed or vice versa when left-handed; corrections were allowed to be made within the allocated time. Performance was determined by the total amount of pinholes filled correctly. The number of incorrectly filled holes (i.e., 1–2, or >3 pins·hole^−1^) was also recorded.

Cognitive performance was assessed with the Spaceflight Cognitive Assessment Tool for Windows (WinSCAT; Wyle Integrated Science and Engineering Group, Houston, TX; Ref. 84). This computer-based tool encompasses five cognitive subtests: *1*) the code substitution subtest, which assesses learning; *2*) the running memory subtest, which assesses working memory, concentration, and attention; *3*) the mathematical processing subtest, which assesses working memory, concentration, and mathematical skills; *4*) the match to sample subtest, which assesses spatial processing and visuospatial working memory; and *5*) the delayed code substitution subtest, which assesses short-term memory. For each subtest, accuracy (in the percentage of the correct answer), reaction time (in ms), and throughput (i.e., the number of correct responses per time unit) were measured. A composite score (in arbitrary units), indicating an overall cognitive performance, was also derived from the throughput scores of four subtests (i.e., running memory, mathematical processing, match to sample, and the delayed code substitution subtests).

During each preimmersion phase and immediately after the completion of the cognitive function test performed during CWIs, subjects rated their whole body thermal sensation (from 1 “cold” to 7 “hot”) and comfort (from 1 “comfortable” to 4 “very uncomfortable*”*) (49).

### Data and Statistical Analyses

Data from the baseline phase of the two main sessions did not differ, and thus, for brevity reasons, they were clamped together. The WinSCAT-derived data for AIR CWI_A_ and N_2_O CWI_B_ and the physiological data for N_2_O CWI_B_ were presented for 13 subjects because of technical failure. All data were analyzed with linear mixed-effect models with subjects as a random effect. To evaluate intrasession differences from baseline values, a model was developed for each breathing condition, in which the measurement phase (i.e., baseline vs. CWI_A_ vs. CWI_B_ vs. CWI_C_) was the mixed effect. To assess intersession differences, a separate model was developed, wherein the breathing condition (i.e., AIR vs. N_2_O) and CWIs (i.e., CWI_A_ vs. CWI_B_ vs. CWI_C_) were the mixed effects. For all models, the best-fitting covariance structure was assessed with Hurvich and Tsai’s criterion. When significant *F* ratios were noted, post hoc comparisons were made with Bonferroni-adjusted *P* values. The within-day (using data from the last 3 trials in the preliminary session) test-retest reliability for the finger dexterity and the cognitive function tests was assessed with the intraclass correlation coefficient (ICC) and the coefficient of variation (CV). The internal consistency of the POMS-SF and MFI subscales was examined with the Cronbach’s α-coefficient. Statistical analyses were performed using SPSS 27.0 (IBM SPSS Statistics for Windows, Armonk, NY) and Prism 9.0 (Graphpad Software, Inc., San Diego, CA). Unless otherwise stated, data are presented as mean values with standard deviation. Statistical significance was set a priori at 0.05.

## RESULTS

The mean (range) onset time for the psychometric and cognitive testing was at *minutes 63* (31–70) and *61* (38–73) in the AIR and N_2_O sessions, respectively (*P* = 0.14). During the period of testing, T_rec_ and T_sk_ were, on average, reduced by ∼1.2°C and ∼8°C, respectively (*P* < 0.001; Table 1). N_2_O aggravated the T_rec_ drop in CWI_A_ and CWI_C_ (*P* < 0.001) but not in CWI_B_ (*P* = 0.83). T_sk_ was lower by ∼0.4°C in N_2_O than in AIR CWI_C_ (*P* = 0.04). N_2_O blunted V̇o_2_, V̇_I_, and HR in all CWIs (*P* < 0.001), and the mean arterial pressure in CWI_A_ and CWI_B_ (*P* = 0.02) (Table 1). The cold-evoked reduction (*P* ≤ 0.05) in Δ[O_2_Hb] and Δ[tHb] was attenuated by N_2_O in CWI_C_ (*P* = 0.02; Table 1). During the N_2_O session, the Δ[tHb] drop was less in CWI_B_ and CWI_C_ than in CWI_A_ (*P* ≤ 0.05). The cold-induced elevation (*P* ≤ 0.001) in Δ[HHb] was slightly greater in N_2_O than in AIR CWI_C_ (*P* ≤ 0.01).

**Table 1.** Physiological responses obtained during the three repeated cold-water immersions while subjects were breathing either room air or a normoxic gas mixture containing 30% nitrous oxide

	Baseline	CWI_A_		CWI_B_		CWI_C_	
		Air	N_2_O	Air	N_2_O	Air	N_2_O
T_rec_, °C	37.2 (0.2)	36.2 (0.5)†	35.8 (0.6)†*	36.0 (0.5)†	36.0 (0.5)†	36.0 (0.5)†	35.7 (0.3)†*#
T_sk_, °C	32.7 (0.6)	24.5 (0.5)†	25.0 (1.1)†	24.5 (0.4)†	24.3 (0.8)†	24.8 (0.6)†	24.4 (0.5)†*
${\bar{\text{T}}}_{\text{b}}$, °C	35.6 (0.3)	31.9 (0.5)†	31.8 (0.7)†	31.9 (0.4)†	31.7 (0.7)†	32.0 (0.4)†	31.7 (0.2)†*
V̇o_2_, L·min^−1^	0.34 (0.07)	0.64 (0.20)†	0.48 (0.19)*	0.59 (0.22)†	0.39 (0.10)*	0.60 (0.25)†	0.42 (0.14)*
V̇_I_, L·min^−1^	8.3 (1.1)	13.6 (6.0)†	10.5 (3.9)*	12.8 (5.2)†	8.8 (2.4)*	13.8 (5.4)†	9.0 (2.3)*
MAP, mmHg	99 (11)	115 (12)†	108 (13)†*	113 (11)†	105 (16)*	112 (11)†	113 (12)†
HR, beats·min^−1^	70 (12)	64 (10)†	57 (9)†*	65 (11)	56 (10)†*	67 (9)	56 (10)†*
Δ[O_2_Hb], μM	−1.3 (3.3)	−15.9 (7.8)†	−17.7 (7.8)†	−14.8 (6.7)†	−11.4 (7.5)†	−15.7 (7.0)†	−10.8 (6.1)†*
Δ[HHb], μM	−0.9 (1.8)	2.9 (2.8)†	2.4 (2.2)†	3.1 (2.8)†	1.7 (3.8)†	1.0 (2.5)†#	3.6 (2.9)†*
Δ[tHb], μM	−2.2 (4.1)	−13.0 (8.0)†	−15.3 (8.2)†	−11.7 (6.0)†	−9.7 (8.2)†§	−14.7 (7.1)†	−7.2 (7.6)†*§

Values are mean (SD) for rectal temperature (T_rec_), skin temperature (T_sk_), mean body temperature (${\bar{\text{T}}}_{\text{b}}$), oxygen uptake (V̇o_2_), inspiratory minute ventilation (V̇_I_), mean arterial pressure (MAP), heart rate (HR), and changes (from the 3-min baseline phase; see *Experimental Protocol*) in oxygenated (Δ[O_2_Hb]), deoxygenated (Δ[HHb]), and total hemoglobin (Δ[tHb]). Baseline recordings were performed in room-air breathing conditions (*n* = 14 men; except for N_2_O CWI_B_ where *n* = 13). Data were analyzed with linear mixed-effect models with best-fit covariance structure, followed by Bonferroni post hoc testing (*P* < 0.05). AIR, normal air; CWI_A_, 1st cold-water immersion; CWI_B_, 2nd cold-water immersion; CWI_C_, 3rd cold-water immersion; N_2_O, nitrous oxide. †Significantly different from baseline. *Significantly different from AIR. §Significantly different from CWI_A_. #Significantly different from CWI_B_.

### Mood State and Thermoperception

**Figure 2.**
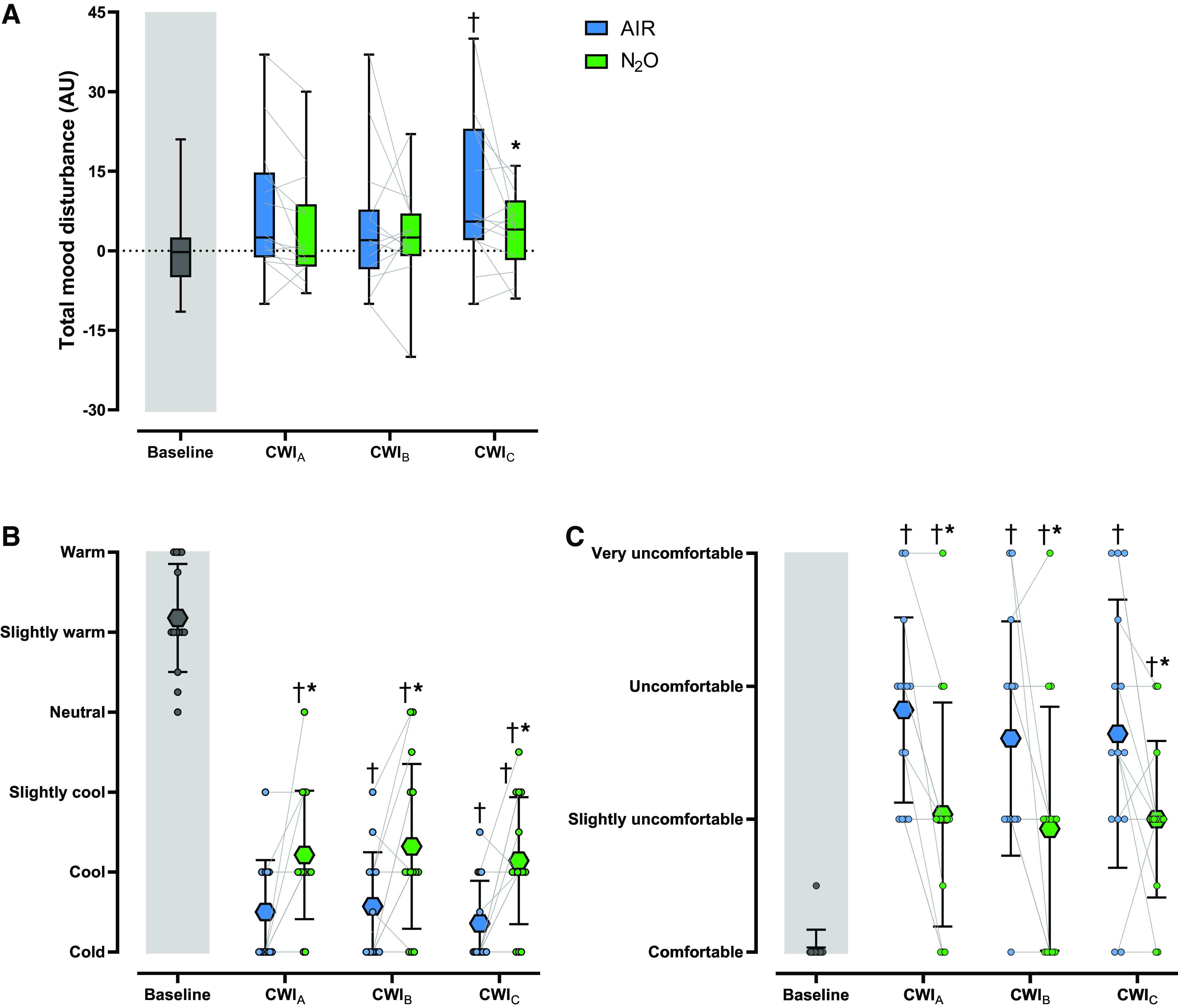
Box and whiskers plot (5–95% percentile) and individual values of total mood disturbance derived from the Profile of Mood States-Short Form (*A*) and mean (SD) and individual values of thermal sensation (*B*) and comfort (*C*) obtained during the 3 repeated cold-water immersions (CWI_A_, 1st cold-water immersion; CWI_B_, 2nd cold-water immersion; CWI_C_, 3rd cold-water immersion), while subjects (*n* = 14 men) were breathing either room air (AIR) or a normoxic gas mixture containing 30% nitrous oxide (N_2_O). All baseline recordings were performed in room-air breathing conditions. AU, arbitrary units. Data were analyzed with linear mixed-effect models with best-fit covariance structure, followed by Bonferroni post hoc testing (*P* < 0.05). †Significantly different from baseline. *Significantly different from AIR.

The mean values of total mood disturbance and each subscale derived from POMS-SF are presented in Fig. 2*A* and Table 2, respectively. Overall, the subjects’ mood was more disturbed in AIR than in N_2_O CWIs; the response was more prominent in CWI_C_ (*P* < 0.01; Fig. 2*A*). During the AIR session, vigor was impaired throughout (*P* < 0.01), and, in CWI_C_, the sensation of fatigue was compounded (*P* ≤ 0.02) (Table 2). N_2_O evoked less impairment in vigor (*P* < 0.01) but enhanced confusion (*P* = 0.03) during CWI_A_. The self-reported tension and fatigue were also diminished in N_2_O CWI_C_ (*P* = 0.03).

**Table 2.** Mean (SD) values of the Profile of Mood States-Short Form and the Multidimensional Fatigue Inventory subscales obtained during the three repeated cold-water immersions while subjects were breathing either room air or a normoxic gas mixture containing 30% nitrous oxide

	Cronbach’s α-Coefficient	Baseline	CWI_A_		CWI_B_		CWI_C_	
			Air	N_2_O	Air	N_2_O	Air	N_2_O
POMS-SF								
Tension	0.79	2.3 (3.4)	3.1 (3.4)	1.8 (2.4)	3.1 (3.4)	1.0 (1.3)	3.1 (3.1)	0.9 (1.4)*
Depression	0.78	0.7 (1.6)	2.3 (3.5)	1.9 (2.3)	1.4 (2.7)	1.3 (2.0)	1.8 (3.0)	0.2 (0.4)§
Vigor	0.85	8.1 (4.5)	3.9 (4.4)†	6.9 (4.2)*	4.5 (4.5)†	4.9 (5.7)†	3.6 (4.4)†	4.0 (3.7)†§
Anger	0.76	0.8 (1.9)	1.7 (2.2)	1.0 (2.5)	1.2 (2.2)	0.3 (0.6)	1.1 (1.7)	0.4 (0.8)
Confusion	0.72	1.4 (1.8)	1.6 (1.9)	3.4 (3.2)*	1.7 (3.1)	1.8 (1.6)	2.4 (2.9)	2.7 (2.2)
Fatigue	0.76	3.2 (2.1)	2.7 (2.3)	2.5 (2.5)	3.1 (2.6)	3.0 (2.9)	5.4 (4.5)§#	3.6 (3.4)*
MFI								
General fatigue	0.68	9.4 (2.9)	10.6 (3.5)	9.4 (3.5)	12.0 (3.6)†	11.6 (3.3)§	12.9 (3.4)†§	11.1 (3.5)*
Physical fatigue	0.85	7.7 (3.1)	10.0 (4.7)†	8.8 (3.7)	9.0 (4.5)	8.1 (3.1)	10.6 (4.0)†	9.3 (3.8)
Reduced activation	0.76	9.7 (4.0)	11.1 (3.4)	10.7 (4.0)	10.9 (4.3)	10.3 (3.7)	12.1 (4.3)†	10.4 (3.6)*
Reduced motivation	0.56	8.1 (2.3)	10.1 (3.3)	8.9 (2.9)	10.1 (3.4)	9.4 (2.6)	11.6 (3.5)†	8.9 (2.8)*
Mental fatigue	0.84	10.4 (3.9)	11.9 (3.8)	13.2 (4.0)†	12.1 (3.8)†	13.1 (3.5)†	12.1 (4.1)†	13.6 (3.9)†*

Baseline recordings were performed in room-air breathing conditions (*n* = 14 men). Data were analyzed with linear mixed-effect models with best-fit covariance structure, followed by Bonferroni post hoc testing (*P* < 0.05). AIR, normal air; CWI_A_, 1st cold-water immersion; CWI_B_, 2nd cold-water immersion; CWI_C_, 3rd cold-water immersion; N_2_O, nitrous oxide; MFI, Multidimensional Fatigue Inventory; POMS-SF, Profile of Mood States-Short Form. †Significantly different from baseline. *Significantly different from AIR. §Significantly different from CWI_A_. #Significantly different from CWI_B_.

The mean values of MFI subscales are summarized in Table 2. During the AIR session, subjects reported enhanced sensations of general and mental fatigue in CWI_B_ and CWI_C_ (*P* ≤ 0.03), as well as of physical fatigue and reduced activation and motivation in CWI_C_ (*P* ≤ 0.004). N_2_O exacerbated the perceived levels of mental fatigue in all CWIs (*P* < 0.01) but did not elicit other fatigue-related sensations (*P* > 0.05).

N_2_O alleviated the sensation of coldness and thermal discomfort throughout (*P* < 0.001; Fig. 2, *B* and *C*).

### Finger Dexterity

The within-day ICC and CV were 0.94 and 5%, respectively. T_finger_ did not differ between CWIs [AIR session: CWI_A_: 25.2 (1.2)°C; CWI_B_: 24.7 (0.9)°C, CWI_C_: 24.7 (0.7)°C; N_2_O session: CWI_A_: 25.6 (2.9)°C, CWI_B_: 24.3 (3.3)°C, CWI_C_: 24.4 (1.9)°C; *P* = 0.15].

**Figure 3.**
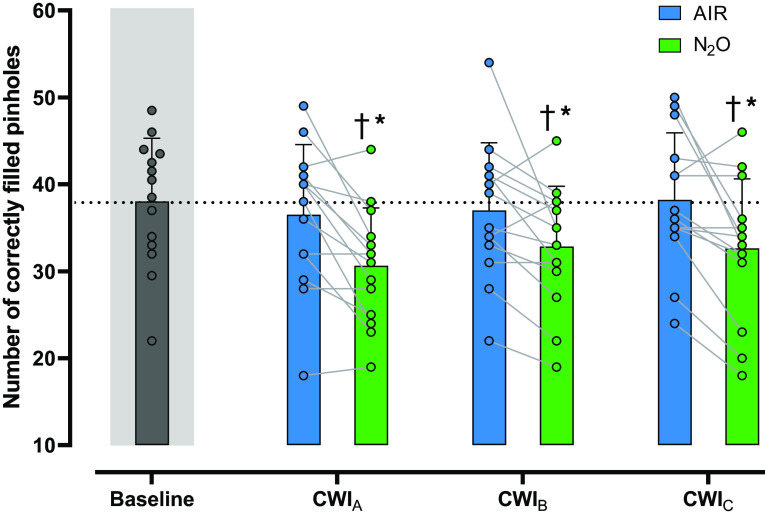
Mean (SD) and individual values of the total number of correctly filled pinholes in the 3-min finger dexterity test performed during the 3 repeated cold-water immersions (CWI_A_, 1st cold-water immersion; CWI_B_, 2nd cold-water immersion; CWI_C_, 3rd cold-water immersion), while subjects (*n* = 14 men) were breathing either room air (AIR) or a normoxic gas mixture containing 30% nitrous oxide (N_2_O). Baseline recordings were performed in room-air breathing conditions. Data were analyzed with linear mixed-effect models with best-fit covariance structure, followed by Bonferroni post hoc testing (*P* < 0.05). †Significantly different from baseline. *Significantly different from AIR.

In the AIR session, finger dexterity did not vary from the baseline phase and remained unaffected across the three CWIs (*P* > 0.05; Fig. 3). By contrast, N_2_O consistently compromised the capacity to correctly fill the pinholes (*P* = 0.001; Fig. 3); subjects were also more prone to make errors, especially in CWI_A_ [the mean (range) number of incorrectly filled pinholes was 0.4 (0–2) in the AIR session and 0.9 (0–5) in the N_2_O session; *P* < 0.01].

### Cognitive Performance

The within-day ICC and CV were 0.92 and 10%, respectively. N_2_O slightly prolonged the duration of the cognitive testing [mean (range) in AIR session: 16 (14–19) min and in N_2_O session: 17 (14–21) min; *P* < 0.001]. On a few occasions in the N_2_O CWIs, the investigators had to mildly encourage subjects to remain focused on the testing, because some of them experienced somnolence.

**Figure 4.**
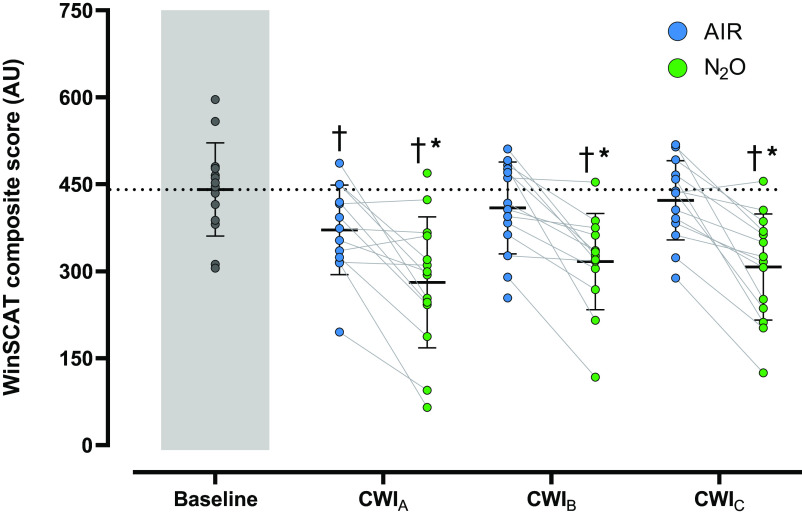
Mean (SD) and individual values of the composite score derived from the cognitive [Spaceflight Cognitive Assessment Tool for Windows (WinSCAT)] test performed during the 3 repeated cold-water immersions (CWI_A_, 1st cold-water immersion; CWI_B_, 2nd cold-water immersion; CWI_C_, 3rd cold-water immersion), while subjects (*n* = 14 men; except for AIR CWI_A_ and N_2_O CWI_B_ where *n* = 13) were breathing either room air (AIR) or a normoxic gas mixture containing 30% nitrous oxide (N_2_O). Baseline recordings were performed in room-air breathing conditions. AU, arbitrary units. Data were analyzed with linear mixed-effect models with best-fit covariance structure, followed by Bonferroni post hoc testing (*P* < 0.05). †Significantly different from baseline. *Significantly different from AIR.

During the AIR session, the overall cognitive performance, as reflected by the composite score (Fig. 4), was impaired by ∼14% in CWI_A_ (*P* = 0.03) but was unaltered in CWI_B_ and CWI_C_ (*P* > 0.05). N_2_O aggravated the cold-induced reduction in cognitive performance during CWI_A_ (*P* < 0.001); contrary to the AIR session, the impairment prevailed across the two repeated N_2_O CWIs (*P* < 0.001).

**Figure 5.**
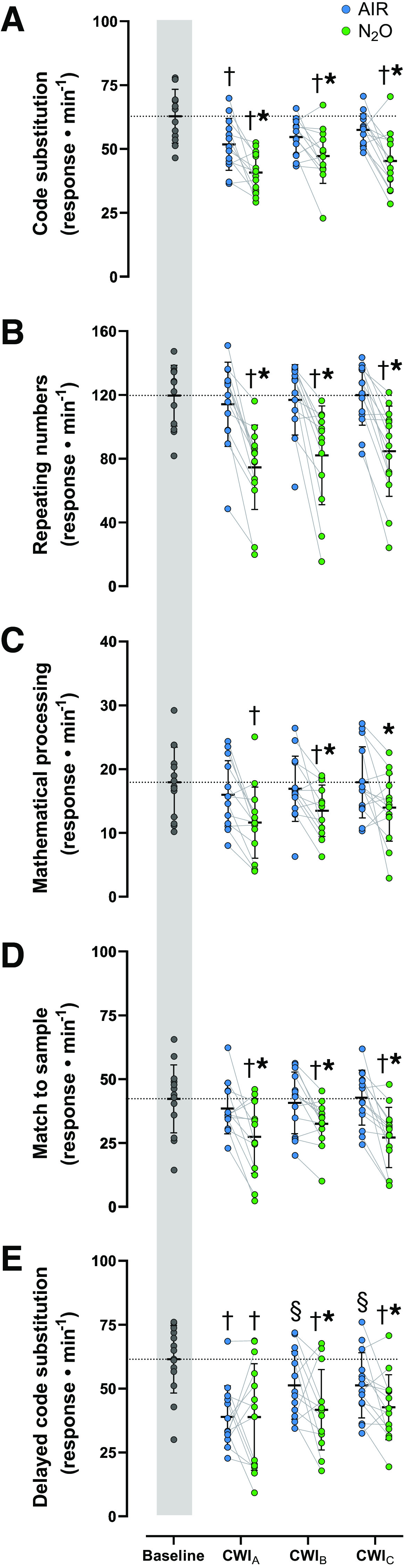
Mean (SD) and individual values of the throughput score for the code substitution (*A*), the running memory (*B*), the mathematical processing (*C*), the match to sample (*D*), and the delayed code substitution (*E*) subtests derived from the cognitive (WinSCAT) test performed during the 3 repeated cold-water immersions (CWI_A_, 1st cold-water immersion; CWI_B_, 2nd cold-water immersion; CWI_C_, 3rd cold-water immersion), while subjects (*n* = 14 men; except for AIR CWI_A_ and N_2_O CWI_B_ where *n* = 13) were breathing either room air (AIR) or a normoxic gas mixture containing 30% nitrous oxide (N_2_O). Baseline recordings were performed in room-air breathing conditions. Data were analyzed with linear mixed-effect models with best-fit covariance structure, followed by Bonferroni post hoc testing (*P* < 0.05). †Significantly different from baseline. *Significantly different from AIR. §Significantly different from CWI_A_.

The mean values of throughput obtained during all cognitive subtests are illustrated in Fig. 5. During the AIR session, only the code substitution and the delayed code substitution were degraded in CWI_A_ (*P* ≤ 0.01), but, in CWI_B_ and CWI_C_, both were reverted toward the baseline values (*P* > 0.05). By contrast, N_2_O invariably impaired all cognitive subtests (*P* ≤ 0.05). Specifically, N_2_O compromised, in all CWIs, the accuracy and the reaction time for the code substitution, the running memory, and the match to sample subtests (*P* ≤ 0.001; Table 3). During the N_2_O subtest of mathematical processing, the accuracy was reduced throughout, but the reaction time increased only in CWI_A_ (*P* < 0.001; Table 3). N_2_O consistently diminished accuracy for the delayed code substitution subtest (*P* < 0.001; Table 3).

**Table 3.** Mean (SD) accuracy and reaction time for each cognitive subtest derived from the cognitive function (WinSCAT) test performed during the three repeated cold-water immersions while subjects were breathing either room air or a normoxic gas mixture containing 30% nitrous oxide

	Baseline	CWI_A_		CWI_B_		CWI_C_	
		Air	N_2_O	Air	N_2_O	Air	N_2_O
Code substitution							
Accuracy, %correct	97 (2)	94 (5)	90 (5)†*	96 (4)	89 (6)†*	96 (4)	90 (7)†*
Reaction time, ms	951 (173)	1,116 (213)	1,368 (290)†*	1,075 (166)	1,211 (404)†	1,012 (126)§	1,263 (342)†*
Running memory							
Accuracy, %correct	93 (5)	90 (10)	75 (18)†*	91 (8)	75 (20)†*	91 (5)	77 (18)†*
Reaction time, ms	472 (66)	478 (68)	560 (67)†*	473 (62)	557 (94)†*	462 (61)	539 (69)†*
Mathematical processing							
Accuracy, %correct	86 (12)	80 (11)	65 (19)†*	84 (14)	73 (18)†*	87 (11)	72 (23)†*
Reaction time, ms	2,973 (625)	3,041 (619)	3,385 (717)*	3,069 (601)	3,227 (632)	2,837 (1,083)	3,062 (662)
Match to sample							
Accuracy, %correct	92 (10)	93 (9)	76 (18)†*	90 (9)	87 (10)§	93 (10)	77 (15)†*#
Reaction time, ms	1,403 (429)	1,519 (355)	1,937 (1,036)†	1,393 (354)	1,647 (429)*	1,356 (342)	1,714 (707)*
Delayed code substitution							
Accuracy, %correct	92 (8)	79 (13)†	63 (12)†*	85 (13)	66 (17)†*	81 (12)†	64 (17)†*
Reaction time, ms	955 (250)	1,261 (239)	1,251 (751)	1,008 (161)§	1,051 (407)	1,009 (325)	1,016 (380)

Baseline recordings were performed in room-air breathing conditions (*n* = 14 men; except for AIR CWI_A_ and N_2_O CWI_B_ where *n* = 13). Data were analyzed with a linear mixed-effect model with best-fit covariance structure, followed by Bonferroni post hoc testing (*P* < 0.05). AIR, normal air; CWI_A_, 1st cold-water immersion; CWI_B_, 2nd cold-water immersion; CWI_C_, 3rd cold-water immersion; N_2_O, nitrous oxide; WinSCAT, Spaceflight Cognitive Assessment Tool for Windows. †Significantly different from baseline. *Significantly different from AIR. §Significantly different from CWI_A._

## DISCUSSION

Short-term cold stress and inert gas narcosis independently jeopardize cognitive and intellectual functions (1, 18). The present study, therefore, aimed to investigate, in healthy men, the interactive influence of whole body cooling and narcosis produced by a subanaesthetic dose of N_2_O, on neurocognitive performance during prolonged intermittent exposure. To this end, basic (i.e., thermoperception and fine motor skills) and higher-order (i.e., aspects of attention, memory, learning, and visuospatial processing) cognitive abilities were evaluated repeatedly within a ∼10-h period, while subjects were rendered mildly hypothermic (i.e., ∼1.2°C drop in T_rec_) by means of three serial CWIs and were inhaling either normal air or 30% N_2_O. The main findings were *1*) cooling per se temporarily degraded (i.e., only in CWI_A_) selective cognitive abilities, that is, short-term memory and learning; and *2*) superimposition of N_2_O-induced narcosis on cold strain aggravated, in a persistent manner, all cognitive properties assessed.

### Effects of Cold on Cognition

The acute cold provocation compromised by ∼14% the overall cognitive function. Yet, the disturbance was limited to two cognitive features, short-term memory (assessed by the delayed code substitution subtest) and associative learning (assessed by the code substitution subtest), whereas attention, concentration, working memory, and spatial processing appeared to be resistive to mild hypothermia. Previous studies have provided equivocal results with regard to the impact of whole body cooling on aspects of attention and memory: a few have observed impairments (48, 50–53), whereas others observed no changes (48, 52, 54). Also, and similarly to our observation, Payne and Cheung (55) found that the visuospatial abilities remained intact in mildly hypothermic individuals. By the current experimental design, we are unable to identify the mechanisms underlying the selective impact of cold stress on cognition. This, however, cannot be associated with differences in the complexity of subtests performed, because all cognitive operations comprised executive functioning components (cf. Ref. 67). Arguably, in view of the high degrees of interindividual variability, the study was powered inadequately (note: a post hoc power analysis revealed that 100–150 subjects would have been required) to reveal the influence of cold stress on all cognitive domains.

Nevertheless, the cold-evoked effects on the two cognitive capacities were transient and abated fully during the two successive CWIs; presumably suggesting a gradual induction of nonassociative learning (i.e., habituation). Such short-term adaptive response seems to be in line with that noted following longer cold-acclimation regimens. For instance, Jones et al. (51) found that seven daily 90-min immersions in 10°C water partly enhanced cognitive function to acute cold stress. This intervention (51) also led to an insulative cold adaptation, described by augmented peripheral vasoconstriction, attenuated shivering, and blunted thermal discomfort. The cognitive habituation obtained in the present study, however, was not accompanied by any thermoadaptive adjustments and apparently was independent of any intrasession variations in the physiological strain (see Table 1). In addition to our measurements, Castellani et al. (57), who utilized a similar experimental design to the present, did not detect any modifications in the secretion of plasma norepinephrine across the three repeated CWIs. It is noteworthy that, in our study, both cognitive abilities were reverted to the preimmersion qualities, despite the prevalence of thermal discomfort, and the gradually compounded levels of perceived fatigue and mood disturbance. We thus speculate that subjects, after the initial shock, were able to tolerate the hypothermic strain by learning somewhat rapidly to ignore the cold-evoked sensory distractions, thereby devoting sufficient amounts of attention to the required cognitive tasks. Arguably, the performance recovery might simply reflect a “learning effect” resulting from the subjects’ familiarization with the specific cognitive test. Such an explanation, however, seems less likely, considering the relatively high reliability and reproducibility of the WinSCAT test (i.e., ICC was estimated to be >0.90 in our laboratory). Also, the magnitude of difference between CWI_A_ and the two repeated CWIs ranged between 25% and 42%, exceeding by far the estimated within-day test variation that corresponded to 10%. Finally, to allow subjects to acquaint themselves thoroughly with the test and in accord with the recommendations from the test’s developers (cf. Ref. 84), subjects were required to perform the WinSCAT test six times during the preliminary session. Still, further work is warranted to explore the neuroanatomical mechanisms that are responsible for the monophasic impairment of selective cognitive outputs to prolonged whole body cooling.

### Effects of N_2_O and Cold on Cognition

In support of our hypothesis, N_2_O interfered with sensory, affective, and intellectual processes, compromising markedly all cognitive domains tested. N_2_O also suppressed autonomic thermoeffector function (i.e., shivering thermogenesis, as indicated by the blunted elevation in V̇o_2_), accelerating the rate of body core cooling; a finding that conforms to those from other works (34–37) and has been discussed extensively in our previous study (38). Considering that, in the present investigation, intersession differences in cognition were assessed by using an open-loop experimental approach (i.e., subjects’ thermal status remained unclamped), it may be reasoned that the augmented cognitive decline in N_2_O was dictated by the more prominent hypothermia obtained in this condition. However, during the period of testing, although T_rec_ was lower by ∼0.3–0.4°C in N_2_O than in AIR CWI_A_ and CWI_C_, it was identical in the two CWI_B_s (see Table 1). Notwithstanding, the impairment in the perceptual and cognitive outputs prevailed almost equivalently across the three repeated N_2_O CWIs, likely suggesting that the cognitive deterioration in the N_2_O provocations incurred irrespectively of any variations in the magnitude of body-temperature displacements. Rather, the response was probably mediated by the direct action of the gas on the central nervous system. It is currently well documented by animal-based research, that N_2_O disrupts the function of synaptic transmission (58), mainly via the inhibition of the *N*-methyl-d-aspartate glutamatergic receptors (56, 59–61), which in turn supresses the release and turnover of dopamine and other catecholamines in selective cerebral structures (i.e., striatum, prefrontal cortex, hippocampus; Refs. 85, 86) that are involved in affective processing, memory, motivation, learning, and executive functioning. Of interest is that, in the present study, the cold-evoked sympathetic arousal was invariably dampened by N_2_O, as indicated by the diminished values of HR, V̇_I_, and partly, mean arterial pressure.

N_2_O accentuated the impairments in cognition, in the face of the attenuated load of distraction generated by exteroceptive and interoceptive sensations (cf. Ref. 87). Thus the thermal alliesthesia (i.e., blunted coldness and discomfort), the conscious awareness (via proprioceptive and visual cues) of the absent or minimal shivering tremor, as well as the less negative generalized affective state, failed to counteract, or at least alleviate the N_2_O-induced cognitive decline. Still, the enhanced sensation of mental fatigue and confusion engendered by N_2_O may have affected higher levels of cognitive control, contributing to performance detriments (64). Mental fatigue may thus compromise executive functions primarily through impairments in dorsolateral prefrontal and anterior cingulate cortices (see Ref. 62). Yet the N_2_O-dependent cognitive reductions were probably not linked to the oxygenation status of the cerebral prefrontal cortex, given that the cold-induced drop in Δ[Ο_2_Hb] and Δ[tHb] was either similar in the two sessions (in CWI_A_ and CWI_B_) or less in N_2_O (in CWI_C_). This response might be attributed to the vasodilatory effects of N_2_O on cerebral vessels (63, 66), the blunted neural activation to the cognitive task, and/or the lower metabolic demands associated with the reduced skeletal muscle shivering activity. The NIRS measurements, however, reflect region-specific oxygenation changes and whether other brain regions may have been influenced differently by N_2_O is unclear.

Aside from reducing advanced intellectual skills, comprising multisynaptic neural pathways, N_2_O consistently hampered the capacity to execute a fine motor task, which is regarded to engage minimal cognitive resources and only a limited number of synapses (67). The impairment emerged even without a shivering tremor compromising motor control (68). Contrary to in N_2_O CWIs, the manual dexterity remained intact across the three AIR CWIs; a finding that is not surprising, given that, in all CWIs, the hands were exposed to room-air conditions (∼27°C) and thus T_finger_ was maintained at ∼24–25°C throughout–even during whole body cold exposure, dexterity is impaired when local skin-temperature is ≤20°C (2). It is therefore reasonable to assume that the motor decline in N_2_O CWIs was orchestrated solely by the gas and its discrete impact on the afferent and/or efferent parts of neuraxis. N_2_O may, for instance, have modulated tactile sensitivity (Ref. 88; note: a subject did complain of finger numbness in CWI_A_, wherein his performance was reduced by 43%) and/or impaired spatial awareness affecting the hand-eye coordination (70). The augmented levels of mental fatigue perceived in N_2_O could also have played some role (47). Furthermore, in view of the finding that the manual-performance drop was manifested not only by the less amount of correctly filled pinholes but also by the somewhat enhanced number of errors performed, it may indicate that the response was primarily of central, rather than of peripheral origin. Of interest in this regard is that peripheral nervous conduction velocity is not altered by short-term N_2_O inhalation (71). It should also be noted that a previous study failed to detect any reduction in finger dexterity in a cohort of euthermic individuals who were subjected acutely to similar amounts (i.e., 30%) of N_2_O (21). The present finding may hence allude to a synergistic (i.e., hyperadditive) mode of stressors’ interaction, at least with respect to their influence on basic cognitive capacities.

The N_2_O-induced narcosis degraded, in a consistent manner, all capacities across the three CWIs, inhibiting the cognitive adaptation that emerged in the AIR session. A number of experimental observations in divers have likewise shown that repeated daily episodes of N_2_ narcosis do not alleviate the cognitive dysfunction induced by acute N_2_ narcosis (65, 72, 73). The inability to develop behavioral tolerance to N_2_O prevails despite the previous evidence that metacognitive processes, critical for learning (74), are probably undisturbed by inert-gas narcosis (75). Interestingly, when we informally interrogated the subjects, during the rewarming, asking them to retrospectively reflect on their performance, several of them reported that, in the N_2_O condition, they were aware of making errors while performing the tasks, but, at the same time, they felt incapable to implement the necessary corrections.

Recurring evidence suggests that the narcotic action of N_2_O does not decay promptly after gas withdrawal and residual effects may last for several minutes (i.e., approximately ≤30 min; Refs. 31, 89). A similar response pattern has been observed also after acute hyperbaric N_2_ narcosis, wherein mild cerebral impairments are maintained for ∼30 min postresurfacing (69). The subjects in our study were reinstated to room-air breathing conditions for, at least 100 min during the successive N_2_O exposures, which plausibly was an adequate period for dissipation of the previous gas effects. Yet, even if this was the case, the repetitive gas administration failed to evoke any additive effects, since the magnitude of cognitive decline was alike in the three N_2_O CWIs. We acknowledge, however, that the psychomotor responses might have been influenced partly by a placebo effect, since all, but one subject, were able to identify the gas inhaled. Τo prospectively minimize any expectation bias on the outcome, subjects were made aware of the purpose but not of the directional hypotheses of the study.

Collectively, and regardless of the underlying mechanisms, the present findings, along with our previous result that shivering thermogenesis was impaired persistently over the three repeated cold provocations (38), clearly demonstrate that, in humans, prolonged intermittent exposure to subanesthetic amounts of N_2_O does not elicit autonomic, perceptual, and cognitive adaptation.

### Methodological Considerations

Considering that mood state and cognitive performance are modulated by circadian rhythmicity and the time awake (76, 77), and because, in this study, neither the individual chronotype of the subjects nor the amount of night sleep preceding testing was assessed, we cannot exclude that our results may have been confounded, at least to some extent, by the different timing that the tests were performed across each session/day, and the early morning awakening (subjects reported to the laboratory at 0700). Nevertheless, such diurnal influences, if any, were restricted to the AIR session, whereas obviously they were overridden by narcosis in the N_2_O session. Furthermore, blood glucose dropped slightly during the course of all CWIs, yet none of the subjects suffered from hypoglycemia (glucose was ≥4.0 mmol·L^−1^ throughout; these data are presented in Ref. 38). Also, we cannot rule out that a degree of “respondent fatigue” (78) may have influenced the outcome of POMS and MFI questionnaires in CWI_C_.

The study would have benefited from the inclusion of an additional (control) session, wherein cognitive function would have been assessed in thermoneutral N_2_O conditions. We, hence, are unable to determine confidently the mode of stressors’ interaction (e.g., additive, synergistic, or antagonistic). Also, since the type and magnitude of an adaptive response are governed by the volume of stress imposed, and especially since the narcotic potency of acute N_2_O inhalation displays a dose dependency (79), it remains to be settled if behavioral tolerance to inert gas narcosis may ensue from different N_2_O dosages, with regards to the partial pressure of, and/or the exposure frequency/duration to gas. It should, however, be borne in mind that the risk for neurotoxicity is augmented by prolonged sustained, or severe N_2_O administration (80). Finally, future work should evaluate whether the combined effects of inert-gas narcosis and mild hypothermia on cognition are sex dependent. Yet, the examination of each stressor separately suggests that sex-related differences may exist in response to cold (81) but probably not to N_2_O (27, 28, 30).

### Perspectives and Significance

In conclusion, the present findings demonstrate that, in healthy men, a subanaesthetic dose of N_2_O exacerbates the neurocognitive deterioration provoked by cold stress. Thus prolonged iterative whole body cooling compromises temporally selective cognitive aspects, such as short-term memory and associative learning. Superimposition of N_2_O-induced narcosis on the cold strain, however, impairs, profoundly and persistently, both basic (i.e., discriminative and affective thermoperceptions and fine motor skills) and higher-order (i.e., attention and concentration, short-term and working memory, learning, and visuospatial processing) cognitive abilities. Our current and previous (38) results, therefore, substantiate that inert-gas narcosis and cold stress impinge, interactively, on physiological, intellectual, and behavioral processes. Thus, during short- and long-term underwater activities, this stressor combination may attenuate operational effectiveness, and, perhaps, threaten survivability (cf. Ref. 82). Information regarding the combined effects of hypothermia and inert-gas narcosis might be useful when tailoring safe operational strategies for divers.

## DATA AVAILABILITY

Data will be made available upon reasonable request.

## GRANTS

The study was funded by the Swedish Armed Forces (Grant No. AF. 9220919); M.E.K. was supported by a salary grant from the KTH Royal Institute of Technology (Grant No. C-2020-0748).

## DISCLOSURES

No conflicts of interest, financial or otherwise, are declared by the authors.

## AUTHOR CONTRIBUTIONS

M.I.M., A.E., M.G., and M.E.K. conceived and designed research; M.I.M., A.E., and M.E.K. performed experiments; M.I.M. analyzed data; M.I.M. and M.E.K. interpreted results of experiments; M.I.M. prepared figures; M.I.M. drafted manuscript; M.I.M. and M.E.K. edited and revised manuscript; M.I.M., A.E., M.G., and M.E.K. approved final version of manuscript.
